# Crisscrossing scapes in the global flow of elite mainland Chinese students

**DOI:** 10.1007/s10734-023-01023-x

**Published:** 2023-04-10

**Authors:** Etienne Woo, Ling Wang

**Affiliations:** 1 Faculty of Education, University of Cambridge, 184 Hills Road, Cambridge, UK; 2 Faculty of Education, University of Hong Kong, Pok Fu Lam Rd, Hong Kong, China

**Keywords:** International student mobility, Chinese student mobility, Appadurai’s scapes, Global flows, Higher education, Globalisation

## Abstract

This paper applies Appadurai’s notion of scapes in globalisation to study international student mobility. Thirty mainland Chinese students were interviewed; the majority of whom studied at prestigious institutions in the West before enrolling in their current PhD programmes at a research-intensive university in Hong Kong (HK) in the immediate aftermath of HK’s large-scale social protests and amidst the Covid-19 pandemic. We seek to understand why these students relocated to HK to further their studies given these turbulent circumstances and how their mainlander identity and sojourns in the West influence their perceptions of HK’s social movements from the perspectives of ethnoscape and ideoscape, respectively. Our findings reveal that HK represented the ‘best’ compromise for our participants, mitigating their nostalgia for home (i.e. mainland China) whilst offering a superior education to the Chinese mainland. Most participants perceived HK as a nationalistic ideoscape, wherein HK people’s pursuit of autonomy is subordinated to the putative Chinese national interests. Moreover, ethnoscape and ideoscape dynamics were found to crisscross other scapes. Generous scholarships (i.e. financescape) provided additional incentives driving student relocations. The persistent consumption of Chinese social media (techno-mediascape) was found to have resulted in worldview conformity between our participants and the Chinese state.

## Introduction

On 4 October 2022, *South China Morning Post* (SCMP), the flagship English-language newspaper in Hong Kong (HK), ran the headline ‘Hong Kong universities attract a record number of mainland Chinese students’ (SCMP 2022). As these students flocked to HK, the disruption caused by the Covid-19 pandemic as well as the 2019–2020 social demonstrations rapidly receded into the rear-mirror view. Many mainland Chinese students transitioned from pandemic-induced restrictions to become globetrotters once more, resuming a trend and a trajectory of social mobility which was unambiguous prior to the disruptions of the pandemic. Concomitantly, British media proclaimed a triumphant year for British universities, with a record number of mainland Chinese students applying for British undergraduate degrees—a 12% increase from 2021 (Blake 2022).

Media excitement towards international student numbers is a reflection of how international student mobility (ISM) is interdigitated with global economic flows. In a globalised higher education market, where students are seen not just as learners but also as consumers and potential human capital (Mintz 2021), the competition between institutions—underpinned by market logic—for international students becomes increasingly intense. Within this neoliberal-led competition mindset, gains for one country are likely to be interpreted in terms of losses for another. This mindset is also attributed to the binary geographical separation in ISM, traditionally considering mobility as unidirectional from students’ homes to host countries. However, the globalisation of higher education has rendered this view untenable. For example, the growth of transnational higher education in mainland China signifies that students can obtain internationally recognised degrees without physically leaving China, for instance, from branch campuses of Western elite universities, such as the New York University Shanghai campus.

Moreover, even with students moving physically, vertical mobility—moving to a country where universities are regarded as being superior in quality to those of the home countries (Teichler 2017, p. 191)—is now complemented by horizontal mobility, through schemes such as ERASMUS in Europe. Horizontal mobility is also taking place among developing countries, with China and Malaysia being popular study destinations for students originating from developing nations (Cheng 2021). These trends indicate that student mobility has become multi-directional and multi-sited. The changes in (im)mobility catalysed by globalising higher education call for the theoretical model to be reconsidered and the concomitant empirical investigation of ISM.

With a complementary approach, our research seeks to depart from a binary understanding of student mobility by studying mainland students’ mobility at Western elite universities before continuing their doctoral studies at one foremost HK university. As a Chinese territory with a history of 156 years of colonial rule, HK’s location is significant for mainland students, offering different political and educational systems than those which prevail in mainland China. However, these advantages are largely a consequence of HK’s political autonomy, an autonomy which has been severely curtailed with the imposition of the National Security Law in 2020, while subtler forms of control are concurrently taking place across university campuses in the territory (Holz 2022).

The relocation of West-educated mainland students to HK manifests three challenges to traditional ISM studies. First, it challenges the precise categorisation of home and host. The mobility of these students can be conceptualised as home (mainland)—host (the West)—quasi-home/quasi-host (HK), on the basis that HK straddles a liminal home and host. Second, it involves both vertical and horizontal (or reverse) mobility. The journey from China to the West is vertical, whereas the trip from the West to HK can be seen as either horizontal or reverse-vertical. Third, their trajectory involves three or more borders once we consider students who have studied in more than one Western country.

Our explorative study utilises Appadurai’s analytical construct of scapes to investigate and understand the global mobilities of this specific group of students. Scapes are ‘the networks of machines, technologies, organisations, texts and actors that constitute various interconnected nodes along which flows can be relayed’ (Urry 2010, p. 355). In Appadurai’s view, globalisation is underpinned by five specific scapes: people, technology, ideas, money and media. Moreover, disjunctures—different logics of flows—ensure the right conditions for global flows. Pushing Appadurai’s model further, we seek to demonstrate that in addition to disjunctures, the scapes also crisscross one another.

## Context: the pandemic, the city on edge and the campus on fire

The years 2019–2021 were tumultuous for ISM, not least for HK, as ISM faced two significant threats. The global health crisis caused by the pandemic has led all stakeholders of ISM worldwide to reimagine the academic mobility that had hitherto seemed inexorable. HK-based universities may have unwittingly reaped certain benefits since the Covid-19 outbreak, as travel bans, visa restrictions and campus lockdowns in the West made travelling cumbersome and parents more hesitant to expose their children to enhanced risk abroad (Mok et al. 2021). However, a potential countervailing factor possibly pushing students away from HK should also be considered. A study of regional student mobility discovered that the host country’s political stability was a critical pull factor for students (Badoo 2021). This was vindicated in HK by the fleeing of international students, as protests shifted from the streets to universities (Kuo 2019a).

From early 2019 to the onset of the pandemic in 2020, HK was a ‘city on edge’ (Hung 2022), roiled by the helter–skelter of political and social changes. ‘Society-wide uprising demanding full democracy’ (Lee 2022, p. 1) unravelled in the forms of widespread peaceful protests, violent clashes with the police, mass arrests, vandalism directed at pro-China businesses, and civil-war-like stand-offs between students and the police at several university campuses. This influences ISM regarding both global concerns and local socio-political factors. It is against this complex and contentious backdrop that our research was conducted.

## Previous research

Mainland students’ experiences in HK have been analysed quantitatively and qualitatively. Based on a survey of 537 research students in one HK university, Zeng (2021) highlighted that mainland students exhibited significantly higher satisfaction with the university’s infrastructure, intellectual and social climate, and peer support service than did local students. Similarly, by surveying 482 local and non-local students, Jung (2019) revealed that while mainland doctoral students ‘were stressed about their perceived competency and by research- and resource-oriented cultures’ (p. 178), the high level of professionalism characterising their relationship with supervisors had moderating effects on these perceptions. As opposed to the higher satisfaction rate of research students identified in previous quantitative studies, qualitative studies focusing on the lived experience of mainland students revealed significant academic challenges when these students migrate to HK’s universities. Linguistic differences, social networks and discrimination were the most noteworthy acculturative stressors in adapting to academic and social life in HK (Yu and Zhang 2016).

An implicit factor underlying these stressors was the fuzzy identity of mainland students, who were perceived as a third identity, being insiders and outsiders simultaneously while living in HK. The mainland identity was found to be inherently linked to political, social and cultural socialisation. As a nation within a state, HK represents a distinct Bourdieusian social and cultural field from mainland China, meaning that a ‘habitus-field’ disjuncture becomes unavoidable (Xu 2017). The rich social, cultural and economic capital possessed in the mainland context does not correspond to advantages in higher education or the job market in HK. The political field is clearly more challenging to navigate than the social and economic fields, since it calls into question students’ ingrained political beliefs and orientations, built up over many years of socialisation on the mainland. Differences in language, identity and habitus inevitably manifest as psychological segregation between local students and those from the mainland, which is exacerbated by fragmented everyday living spaces, closely guarded interpersonal distance and the politicised online space (Tian 2019).

Qualitative studies have identified critical issues reflecting the experiences of mainland students studying at HK universities, such as identity ambivalence, spatial segregation, and field and habitus disjuncture. These issues correspond to mainland students’ predicament of being ‘stuck in the middle’ (Wang and Woo 2022) between local and international students. However, their scope falls into the broader category of acculturation in ISM—cultural, social and academic—irrespective of the particular focus or theoretical framework of corresponding studies. Moreover, these studies privilege individual agency or the structure within which students are situated. We argue that the theoretical perspective should be broadened and a more multi-faceted model utilised for multi-sited and multi-directional ISM. In our study, this multi-faceted model requires us to de-emphasise agency and structure by looking at the fluidity and intersectionality of the flows of people, money, technology, media and ideas across multiple boundaries.

## Analytical springboard

The cultural anthropologist Appadurai coined the concept of ‘scapes’ as a frame by which to understand contemporary cultural flows that occur via the global intersection of people, media, technology, finance and ideologies. Appadurai’s locution scapes are ‘deeply perspectival constructs, inflected very much by the historical, linguistic and political situatedness of different sorts of actors’ (Appadurai 1990, p. 296). His five landscapes of global flow cover ethnoscapes, technoscapes, mediascapes, ideoscapes and financescapes. They reference the topography of people’s mobility, the global reconfiguration of technology, the distribution and dissemination of information, the concatenation of ideas, concepts and ideologies, and the disposition of capital (During 1999). These constructs are expected to capture the ‘complex, overlapping, and disjunctive order’ of global flows (Appadurai 1990, p. 296). The ‘chaotic nature’ of these flows between scapes is known as disjunctures, whereby the existence of scapes and disjunctures provides the conditions for global flows (Appadurai 1999).

In more familiar terms, Appadurai contends that global flows occurring within these five landscapes have their own rationale and logic. According to him, ‘the global relationship’ between these scapes is ‘deeply disjunctive and profoundly unpredictable’, ‘…since each of these landscapes is subject to its own constraints and incentives (some political, some informational and some techno-environmental) at the same time as each acts as a constraint and a parameter for movements in the other’ (Appadurai 1990, p. 298). Therefore, heterogeneity, as opposed to homogeneity, arises in light of globalisation. The concepts of scapes and disjunctures are valuable when investigating the global flow of ISM as they ‘supersede standard geographical thinking in social-cultural analysis’, (Heyman and Campbell 2009, p. 131) and overcome the duality of agency-versus-structure analysis in various ISM mobility studies. While continuing to recognise the importance of agency and structure in students’ mobilities, Appadurai’s model draws our attention to the prominence of flows, thereby uncovering the interaction between people and mobility.

Appadurai coined the scapes in 1990, at the end of the Cold War and the nascent stage of globalisation. Unsurprisingly, his five scapes and disjunctures now appear commonsensical. Still, the perspectival lens is relevant, but the explanatory power ebbs away in an age of globalisation that ‘entails a radical unsettling of the boundaries of social life’ (Chouliaraki and Fairclough 1999, p. 83). Globalisation can be considered through the lens of metaphors of ‘networks and fluid’ that entail ‘complex and enduring connections across space and through time between people and things’ (Urry 2010, p. 354). Given the rise of new technologies and multi-directional worldwide mobilities, intersecting scapes are more emblematic of the globalising ISM we are witnessing. Utilising Appadurai’s conceptualisation of scapes as an analytical springboard to analyse mainland Chinese students’ global mobility in view of the pandemic and social protests in HK, we seek to demonstrate that mobility takes place not only on scapes and through disjunctures but also at the intersections of the scapes.

## Research questions

This paper limits the analytical focus to only certain scapes, enabling us to dive into them in greater detail. Individuals are at the heart of the basic level of global flows insofar as the global landscape is ultimately navigated by agents, who simultaneously experience and constitute a larger aggregated formation of these flows (Appadurai 1990). Accordingly, individuals or the diaspora communities they form can be used as analytical units in this investigation. In our study, our participants represent this kind of community. Hence, the study will focus solely on the flow of people and the ideologies they carry with them. When listing the findings, we will discuss ethnoscape and ideoscape. However, we will also explore other scapes to demonstrate the crisscrossing, embedded and mutually constitutive nature of these two scapes. The following questions will aid our investigation:

- Ethnoscape: Why did these Western-educated mainland students choose HK instead of continuing their studies in the West or moving back to mainland China for their doctoral degrees amidst the pandemic and aftermath of HK’s social unrest?
- Ideoscape: How did they find HK’s social unrest? To what extent were their views impacted by their sojourns in the West or their mainlander identity?
- Intersecting scapes: How do other scapes crisscross ethnoscape and ideoscape? What potential consequences does crisscrossing have?

## Methods

The study adopts a qualitative research design with semi-structured interviews and naturalistic observation as our main collection methods. Our overarching goal is to discover how mainland students comprehend their academic, social, cultural and political circumstances regarding the territory they are/were in during their transborder mobilities. Interview participants included 30 PhD students (Table 1); 22 women and 8 men from different academic disciplines at a large public university in HK. This case university is a premiere institution, top-ranked in East Asia for its promotion of internationalisation and global competitiveness. Previous to their enrolment, 27 participants had obtained at least one degree from an elite Western university considered a research-intensive flagship university, such as a Russel Group university in the UK or an Ivy League or ‘Public Ivy’ in the US. In addition, 25 participants were recipients of the most prestigious scholarship offered by our case university.

**Table 1:** List of participants

Academic disciplines	Pseudonym
Humanities	P2, P25
Social Sciences	P4, P6, P9, P10, P11, P13, P14, P15, P16, P17, P18, P19, P20, P21, P22, P23, P26, P27, P28, P29
Science	P1, P3, P8, P24
Engineering	P5, P7, P12, P30

The participants were recruited through a snowball sampling strategy, and the number of participants in each cohort varied. The 2020 cohort (13 Year 2 participants) bore the double brunt of social unrest and the pandemic. When the 2021 cohort (12 Year 1 participants) applied to our case university in 2020, the pandemic remained persistent as an ongoing global risk, and the shadows of unrest were still lingering. Thus, event and time specificities had a disproportionate effect on these two cohorts, both physically and emotionally. Consequently, their views and experiences were considered to be the most relevant to our research focus. We interviewed a few students from the 2019 cohort (2 Year 3 participants) and 2018 cohort (3 Year 4 participants), whose views served as triangulation to those expressed by the 2020 and 2021 cohorts. All participants were born or raised in mainland China and identified themselves as mainlanders.

Thirty in-depth semi-structured interviews conducted in Mandarin—each lasting an average of one hour—were carried out from August to December 2021 by one of the authors, who was pursuing a PhD at our case university. Interview participants were encouraged to express their views and perceptions of their academic, cultural and social life in the mainland, the West and HK, as well as give their reflections on the perceived similarities and differences of these locations with respect to the aforementioned aspects. Throughout the interviews, the interviewer, acting as a ‘complete observer’, carried out naturalistic observations at the campus and in mainland students’ social media groups (Cohen et al. 2018, p. 543) to gain further insights into participants’ perceptions, concerns, experiences and daily activities. Observation notes helped the researchers find explanations for their findings that supplemented those obtained from the interviews.

All interviews were transcribed verbatim and analysed using NVivo 12. An inductive analysis of the interview data and observation notes was undertaken following Appadurai’s notions of scapes in order to capture the global flow of these students as they traversed multiple borders. A second-level coding phase was conducted to collate similar codes into interrelated categories and themes through ‘selecting, focusing, simplifying, abstracting and transforming’ (Miles and Huberman 1994, p. 10). Intercoder reliability was achieved by adhering to the guidelines provided by O’Connor and Joffe (2020). For each stage of data analysis, the two researchers coded the data independently before comparing their codes. Differences in coding and areas that needed clarification were thus identified, with a consensus being reached after candid discussions between the researchers.

## Findings

### Ethnoscapes: stratification of flows

Regarding mainland students seeking tertiary education in HK, 2020 was an unfortunate year. The 2019–2020 social unrest pushed university campuses to the epicentre of the turmoil; subsequently, the Covid-19 pandemic hit. When writing (early September 2022), a miasma of restrictive Covid social-control policies remained in HK; it was significant for us to see how these events affected our participants’ decision to move to HK. Concomitantly, if these factors were negative enough for Year 1 and Year 2 students to steer clear of HK, they would not have applied to our case university in the first place. To restate, the pull factors examined below outweighed the potential push factors. Year 1 and Year 2 students did not consider the protests too dangerous to threaten their studies or personal safety.

Participants usually came up with several pros and cons for choosing our case university or HK when asked why they chose HK instead of pursuing their degrees in the West. In this verbal weighing process, no participant mentioned HK social movements as a drawback. One participant who studied colonial history said these protests were attractive to her since they offered a window of opportunity to observe post-colonial social transformation (P2). We learnt that the progressive de-escalation starting in early 2020 thanks to forceful police intervention and the pandemic, the belief in the rule of law in HK and the envisaged passage of the National Security Law in mid-2020 contributed to their confidence in the restoration of stability in HK. Throughout our interviews with Year 1 and Year 2 students, the influence of the protests on their decision to move to HK was found to be negligible, whereas the pandemic had greater resonance with our participants’ motivations and concerns underpinning their decisions to relocate. The negligible effect of the protests on their decision-making process came as a surprise to us because the negative portrayal in Chinese official state media and (state-controlled or state-influenced) social media was deemed to be potentially pivotal. The protests which swept across HK were relatively well reported, receiving substantial coverage across the Chinese state and social media, with a consistent tone which sharply criticised protesters who were caricatured as unpatriotic rioters. China’s state broadcaster, CCTV, blamed the protest movement on the machinations of ‘foreign powers’ in their feature-length documentary entitled *Another Hong Kong* in May 2020 (Vickers and Morris 2022). The image of HK as a chaotic place disrupted by unruly rioters was also popularised throughout Chinese social media and found a wide audience among the Chinese diaspora (Luqiu and Kang 2021). Of the 25 Year 1 and Year 2 students whose decision to study in HK may have been impacted by the then-ongoing pandemic, only four participants identified it as the sole factor that made them come to HK. A dozen of the 25 students listed the pandemic as one factor among several others, including scholarship, compatibility with supervisors, the relative ease of applying to our case university compared to Western elite universities, and the degree quality at this university influencing their decision to relocate to HK. Hence, the role of the pandemic was more moderating than meditating.

For both the dozen participants mentioned above and those who did not mention the pandemic as a reason, being ‘closer to home,’ ‘tired of living abroad,’ and ‘going back home after their PhD’ were the leading reasons that drew them to HK. Even for participants who relocated to HK solely due to the pandemic, the ostensible rationale provided a sense of urgency for escaping the Covid-ridden West. However, underlying that exigency was a sense of security afforded by HK, whose quasi-home status and competent handling of the pandemic served as both a physical and psychological haven. Four (of 13) Year 2 and two (of 12) Year 1 PhD students had already decided to apply to universities in HK before the pandemic struck. Like the students impacted by the pandemic, they cited being closer to home as the main, if not the only reason for choosing HK. A total of 22 of all 30 participants discussed the notion of inside versus outside. As foreign countries, the West was constantly referred to as *Guowai* (overseas, meaning outside of the country).

They formed a solid attachment to *Guonei* (domestic, meaning inside of the country) when listing their reasons for pursuing doctoral studies in HK. They referred to the mainland as *Guonei*—the equivalent of home—and HK as a location closer to home. The yearning to return home was captured by one Year 3 PhD student who had done her bachelor’s and master’s in the US. Her decision to apply to this university was neither affected by the pandemic (which had not arrived at the time of her decision-making) nor by social protests (which were peaceful):

I had been in America for many years, and then I felt that, in any case, I had learned and gained a lot; but I have also missed a lot, like staying with my family or meeting relatives and friends. So, I wanted to choose a place closer to home (P13).

Moreover, the desire to be close to home was partially influenced by the wishes of family members on the mainland. In a society that remains heavily influenced by collective ideals, opinions from immediate family members are commonly considered when one makes an important decision, as outlined by one married participant:

I am married, and I want to continue my studies. This is already not a simple matter in the Chinese cultural context. It would be too much for me to insist on going abroad and still expect family support. When I proposed HK, all my family members supported me (P14).

Thus, why did these students not return to the mainland for their doctoral studies? Returning to the mainland for their doctoral research did not appear as an option for the participants barring one. They were firm regarding their decision not to pursue their studies in mainland China, as highlighted by one student: ‘I don’t want to go abroad anymore, but I also do not want to do it in the mainland either. Therefore, I chose the midpoint—HK’ (P27). This sharp U-turn occurred because going back to the mainland would conflict with their practical needs. Such needs were made apparent when we asked the participants to rank their overall academic experiences in the mainland, the West, and HK. A significant majority ranked the West as the best, while five voted for HK and only two for the mainland. The US, Europe and HK represented a similar higher education system, while the mainland was completely different (P2). Factors such as the lack of academic freedom in mainland China, the chance to pursue majors or courses in the West, deeply entrenched Confucian social hierarchy, and bureaucratic red tape throughout university administration were the primary reasons for disliking the mainland system. One participant avoided choosing a doctoral supervisor at this university who had completed their undergraduate degree on the mainland, fearing that the supervisor might have internalised notions of a personal hierarchy (P7).

The decision to study in the West prior to coming to HK was already in and of itself a testament to the participants’ recognition of the West as a place of academic excellence. The perception of American academic excellence stood out among the participants, including those who trained in Europe. Mirroring this high standard was the competitiveness of programs in leading US universities, as stated by P5: ‘I cannot get into a top PhD economics department in the US because it is too competitive.’ Despite the UK’s seemingly declining level of education (P19), it remains highly regarded after the US. Twenty-five participants received full scholarships either from our case university or the HK government. One-third of the participants admitted that the generous scholarship was one of the leading pull factors. As P3 enthused, ‘the university just gave me too much money!’ Even for participants whose decision was not based on a scholarship, they were aware that the scholarships gave HK an edge over its Western competitors: ‘PhD scholarships are scarce in the UK, however, much more abundant in HK […] even if you are self-funded, HK is cheaper than the UK’ (P14).

According to these findings, each segment of the global flows in ISM was characterised by different logic and challenges. HK resulted from a compromise between students’ agency to choose a place near home and the current global configurations underpinning the flow of people, for example, the centre-periphery education landscape. Flux and tensions could potentially be more accentuated in the flow of ideas.

### Ideoscapes: the national other & the West

Appadurai (1999) describes his ideoscapes as ‘concatenations of images’ which are ‘often directly political and frequently have to do with the ideologies of the state and the counter-ideologies of movements explicitly oriented to capturing state power or a piece of it’ (p. 224). The 2019–2020 social movements transformed HK into grounds for an ongoing struggle between democracy and authoritarianism—seen from the protestors’ perspective. However, according to Beijing, it was a battlefield where malign foreign-backed dark forces threatened Chinese national sovereignty. Divergent national and sub-national perceptions of protests percolated among and between people from these strata. Even before the protests, spatial and academic segregation between mainland and HK students was widespread, sometimes spilling over into palpable tensions (Yu and Zhang 2016). Our findings herein reveal that this segregation not only persisted, but also became discernibly worse (that is, polarised), because our participants felt that their political orientation and that of their HK peers could not be more different.

Although our participants were recruited through close-knit interpersonal networks—the author who conducted the interviews knew many of the participants personally—we are keenly aware of participants’ proclivity for preference falsifications or reticence to express fully in a research setting where the unequal power dynamics between the interviewer and participant can render a stressful environment for the participant, especially on issues of political nature. Notwithstanding this unavoidable dynamic, we still wanted to gauge participant perceptions towards the HK protest movements, as we believe even their withholding of opinions can yield significant insights, telling us something about their attitudes. Most of our participants did not claim to understand politics and protests in the same way as their HK counterparts. In sharp contrast to many HK students’ activism and mobilization, our participants viewed activism and civil disobedience as the wrong way to voice their demands. Our findings are in line with previous research which has attributed these differences in attitudes to both political cultures, as well as respective civic education contexts in HK and mainland societies (Fairbrother 2003). Despite the fact that we only asked about their perceptions and attitudes, three participants still told us that the topic was too sensitive for them to discuss. Another three participants were quick to state that they were disinterested in politics, in an attempt to close the topic from further exploration. We did not pursue the matter further with them, but it was readily apparent that they did not want to be embroiled in either Chinese nationalism or HK’s anti-mainland sentiments. Almost all of our participants who responded, even those who were tolerant of protests, held predominantly negative views about the mass demonstrations and social unrest on campuses. The protests were described unsympathetically as ‘crap’ *(Lanshi*) (P1), ‘farce’ (*Hunao*) (P8), ‘riot’ (*Baoluan*) (P13), ‘foul atmosphere’ (*Wuyan Zhangqi*) (P10), and the protesters were labelled as ‘HK secessionists’ (*Gangdu*) (P5) or ‘extremists’ (*Jiduan*) (P19). Their views were in line with those of Chinese official media, as reported by one participant:

In 2019, Taiwan and the United States wanted to make HK a base for anti-Chinese activities. Their emphasis was on two systems rather than one country; however, things changed after promulgating the National Security Law. The law was a blow to HK democrats and pan-democrats, who can no longer do whatever they want. Most importantly, Western countries cannot mess with HK again (P10).

Several students were traumatised by campus violence, given that they had never before witnessed such conflict first-hand. Moreover, most participants exhibited discernible violence bias in their discourse, defined as an overrepresentation of violent events relative to what else was happening (Chenoweth 2020). There was hardly any mention of the largely peaceful demonstrations, let alone an understanding or an attempt to make sense of the demands of the protestors and local students. This was also the case for several liberal-minded participants. For instance, one participant sympathised with the exodus of Hong Kong citizens due to the implementation of the National Security Law and considered the locals mistreated. Nevertheless, her mainland-centric undertones were hard to miss:

Most of the locals disapproved of the use of violence to fight for rights. At least the local students around me, after they realised that there were many mainland students like us […] with whom they could conduct a peaceful exchange […], began to reflect on the bias they had toward us, for example, seeing mainlanders as itinerant traders who buy sports shoes and milk powder in HK and sell in the mainland (P2).

Since the West, especially the US, was portrayed in Chinese media and perceived by some participants as the foreign force fuelling the protests, we were interested in what the West meant to them. We thoroughly understand that ‘what the West means in a given context […] depends entirely upon who is invoking the term and for what purpose’ (McNeill 1997, p. 514). We started by discussing their perception of HK. There was a consensus among participants that HK constituted a hybrid of West and East. According to several participants, a crucial element of the West was the legacy of British colonialism. We then probed further on what the West meant to the participants. From our conversation about the protests, we had expected a relatively unfavourable political view of the West, whose governments were seen as the malign and nefarious instigators of social unrest. It turned out this was not the perspective that the participants had focused on.

About a third of participants remained vague about the idea of the West and failed to come up with definite descriptions or analyses of what the West stood for. Another third considered the West as having different cultural traditions (such as festivals), ways of thinking, cuisine, education systems, language and even work ethics. Nine participants offered a short economic and social analysis, like the West running according to a capitalist system with the benefit of the free market and the downside of social inequality. A few participants additionally saw Western values positively, such as the West’s individualistic value system prizing respect for individual freedom. The contrast between voluntary Covid-19 vaccination in the West and mainland China’s intrusive Covid tracing App was given as an example (P12). In light of our participants’ education and cosmopolitan background, we expected some of them to offer a more detailed and concept-oriented political analysis of the political system, rights and civil society. However, this went unmentioned. An aporia existed between the perception of the West’s political involvement in the protests and a lack of political understanding of the West.

HK’s Eastness and Westness are rooted in its Chinese heritage and sovereignty and the underlying legacy of colonialism. The delicate equilibrium of the fusion becomes challenging to maintain when values clash. Values often associated with the West, such as democracy, freedom and civil disobedience, are incompatible with China’s assimilationist agenda and policy, which see protests as spurred by subversive Western ideas. For some of our participants, HK was a nationalistic ideoscape, wherein HK people’s pursuits of autonomy were considered secondary to overarching Chinese national interests. At the same time, the West was a foreign ideoscape whose values were either irrelevant or to be respected, at best.

## Discussion: crisscrossing scapes

It is clear from the findings above that the flows of people and ideas exhibit various logical flows. Appadurai (1990) labels these flows as the disjunctures of the scapes. In the following discussion, we aim to illustrate the crisscrossing nature of the scapes, which was not discussed in detail in Appadurai’s work but deserves greater attention. Ethnoscape and ideoscape inadvertently cross paths with other scapes in a world of unsettled boundaries, thereby giving rise to an intersecting assemblage of scapes that constitute mainland students’ global flow.

### Differentiated ethnoscapes: affective place vs educational space

In 2020, the pandemic was considered a period of ‘sticky and suspended times’ and ‘asynchronous and precarious times’ for mainland students (Wang 2022). Meanwhile, it undoubtedly accelerated the pace of our participants’ relocation to HK, given that it provided a Kairotic moment—a qualitative aspect of time in the Ancient Greek notion of Kairos, such as the right moment or the opportunity-bearing instant. The relocation to HK provided an exhalation—a relief that travelling back home would be shorter, more accessible and less cumbersome. However, it was motivated by more than the desire to escape. Our findings suggest a deeper rationality linking participants affected by the pandemic to those, more than half of the participants, who proved unaffected. The common denominator was the homecoming, manifested by all participants’ foregrounding of *home*, *Guonei* (domestic) and *Guowa*i (overseas). Paradoxically, ‘home’ is perceived as less desirable regarding educational pursuits. Geographer Yi-Fu Tuan’s humanistic understanding of place and space aids our understanding of the different rationales. A place has been ascribed significance by individuals who link the physical space to their cultural or personal identity. Therefore, the site carries an emotional charge (Tuan 2001). A home is a place because of its inexplicable ‘felt quality,’ referring to ‘the obvious fact that places are not just what we see but also what we hear, smell, and touch, even taste’ (Tuan 2007, p. 53). Although HK does not substitute home, it offers students ‘a sense of being not far away from their hometown’ (P10), not just in terms of geographical distance but also given some aspects of the ‘felt quality’ of home, such as the abundance of hometown food in HK. Therefore, HK’s position is skewed to *Nei* (internal) rather than *Wai* (external), deeming it a temporary home away from home.

Student mobility literature has overemphasised cross-cultural socialisation from a deficit perspective or a strength-based model (Brazill 2022). According to our findings, mainland students’ psychological and sociocultural adjustments (Spencer-Oatey and Xiong 2006) or groupism (Tian 2019) in a foreign country should not merely be regarded as the concomitants of acculturation. For example, these frameworks would treat speaking Mandarin Chinese among mainland students either as a manifestation of language deficiency or, in a more positive light, as a social bonding process. According to Tuan, history, geography and language constitute ‘the triune that gives us a profound sense of identity and home’ as it is ‘a storehouse of past usages and events’ (Tuan 2007). Thus, speaking the mother tongue should be construed as providing a ‘home’ for students in a foreign land.

Space is more abstract than a place, as ‘place is security; space is freedom’ (Tuan 2001, p. 3). HK and the West are differentiated and stratified spaces. The West—primarily the US and, to a lesser extent, the UK—are at the pinnacle of the education hierarchy within participants’ outlooks and perceptions. A large-scale survey directed at mainland and HK first-degree seekers conducted in May 2020 indicated that there was a pivot toward Asia—HK, Japan and Taiwan—for those who would carry on with their studies abroad after the pandemic, while the US and the UK still maintained the first and third most popular destinations (Mok et al. 2021). However, the attractiveness of HK as an education space was inseparable from its position as a regional education hub, supported by the coffers of the city and its universities, as indicated in literature and our own finding.

Finance through scholarships as leverage over its Western counterparts to attract mainland students was indicated in our research. ‘The primary public investment by Hong Kong has been in the form of scholarships to attract international students, most of whom come from China’ (Knight 2017, p.54). The ultra-competitiveness of American scholarships and the scarcity of British scholarships were recurring issues when discussing the financing of doctoral degrees. One student who received an offer from Oxbridge admitted that he would not have chosen HK if he had received a scholarship in the UK (P5). Similarly, several participants forfeited their places at top British universities either because they could not secure funding (P10) or because they received a grant from the Chinese Scholarship Council too meagre compared to scholarships in HK (P16).

Additionally, at the height of the pandemic in 2020, universities in HK scurried to grab talented students (mainly mainland students) by allowing those who previously received offers from top global universities to carry out a direct exchange for a place at HK universities accompanied by an irresistible full-package scholarship (Sharma 2020). Several of our participants who enrolled in 2020 benefited from this scheme. The generous scholarship and ease of application gave HK an edge as a preferred destination in light of times of uncertainty. In October 2022, the HK government unveiled a raft of measures to attract foreign talent, such as extra funding for research students. According to Professor Zweig, a China expert, ‘mainlanders will be more comfortable in a post-National Security Law Hong Kong’. So this could be targeted at mainland masters students overseas: rather than going back to China, get them to come to Hong Kong’ (quoted in Sharma 2022). There are key takeaways from Zweig’s comments. First, financescape (capital) is a critical mediating factor in the global flow of international students. Second, ethnoscape is intertwined with ideoscape, which we now turn to.

### Ideoscape stasis flowing through liquid but controlled techno-media

Following Appadurai’s conceptualisation of the ideoscape, ‘the relationship between states and nations is everywhere an embattled one’ (Appadurai 1990, p. 303). This embattled relationship between nation and state manifests itself not only through the segregation between our participants and their HK counterparts in academic and social life, but it was made more visible by our participants’ displeasure towards the protests. Their overwhelmingly negative feelings towards the protests and associating them with chaos reflects a violence bias characteristic of the Chinese state and social media, which ‘blamed the protest movement on the machinations of “foreign powers”’ (Vickers and Morris 2022, p. 195). Additionally, the media discourse considered HK ‘from a paternalistic, “othering” perspective’ (Cheng 2022) while labelling the actions of the movement as subversive to undermine Chinese sovereignty. These beliefs resonated among our participants in a multitude of ways.

A humanities PhD candidate, who spent two years in the US as an undergraduate exchange student and master’s student, expressed his disapproval of Western faculty members at our case university who supported students: ‘While respecting political opinion and position as personal choices, I am still offended by those Western young assistant professors at my university who supported students’ pursuit of so-called freedom; they are democratic morons’ (P25). He thought of their narrowmindedness as being caused by distorted information flow: ‘Various groups have different channels for receiving information, and even though some places (referring to HK or the West) may seem broadly open, they are very closed. People will not even try to compare their sources of information.’ It was correct of the student to point out the contributing role of techno-media in the rise of disinformation and tribalism in many parts of the world.

Still, according to our observations, social media created the gilded cage for mainland students, making them unaware that they were victims of restricted information. Take the example of the multi-functional application WeChat, China’s most popular form of social media. Our participants depended exclusively on WeChat to communicate with their families and friends back in China since all other foreign alternatives are banned in China. Moreover, WeChat’s public accounts serve as an essential source of information, with public profiles that allow individuals and companies to publish content.

Applying media system dependency theory, which hypothesises that media shapes consumers’ understanding of their environment and action, researchers demonstrated that media consumption on WeChat stuck to old media consumption habits. In addition, the indispensability of WeChat enabled the Chinese government to actively control media and propaganda and transcend national boundaries (Luqiu and Kang 2021). A Chinese University of Hong Kong professor described Chinese media, as well as social media’s portrayal of the 2019 HK protests, as propaganda that intercepted ‘a small part of the information, distorting it and magnifying’ (Kuo 2019b). Stories about thuggish riots, Western infiltration, and secessionists jeopardising Chinese sovereignty go viral on WeChat (Luqiu and Kang 2021). Consequently, HK’s alienation from the mainland was labelled as a ‘misrecognition of a shared cultural essence’ fermented by malign Western interference (Vickers and Morris 2022, p. 191) rather than HK citizens fighting for their rights.

In the digital age, the techno-media flow is formless, shapeless, borderless and timeless. With supposed information abundance, we live on ‘informatisation islands,’ as we receive information from media producers targeting groups of like-minded people (Klinenberg 2005). On the other hand, the state’s traditional form of censorship is aided by novel technology such as ‘reverse censorship’—information flooding (Wu 2018) to control the types of information flow. Despite the fluidity of techno-mediascape, it can be contained. When the techno-mediascape crisscrosses the ideoscape, it can either make ideoscape flourish or leave it static.

## Conclusion

Appadurai celebrated globalisation’s heterogeneity, manifested in the forms of the disjunctures between his five scapes, in contrast to the homogenisation perspectives captured in aphorisms like a global village. The scapes are the ‘building blocks’ of ‘imagined worlds’ of the worldwide flow (Appadurai 1999, p. 222). Put differently, the disjunctures (i.e. heterogeneities) between the scapes make up global flows. The deepening of globalisation since Appadurai’s conceptualisation of scapes in the 1990s has transformed ‘the social as society’ into ‘the social as mobility,’ as claimed by Urry (Urry 2010, p. 348). Multi-sited and multi-directional ISM is an embodiment of diverse mobilities. By applying the concepts of ethnoscape and ideoscape to the mobilities of elite mainland students, we demonstrated that their physical mobilities from the mainland to the West and HK were stratified according to participants’ differentiation of place and space. At the same time, their values and understanding of society remains more or less entrenched in (and concordant with) their place of origin, even as they traverse multiple borders. This is demonstrated by the close alignment of our participants’ perceptions of the HK protests with narratives and explanations which were popularised in the Chinese media. Gu (2015) also demonstrates that while Chinese mainland international students studying in the West exhibit greater understanding towards the host culture thanks to their time abroad, they also become more firmly committed to their own culture. We witnessed the flows of finanscape and techno-mediascape crisscrossing and simultaneously constituting ethnoscape and ideoscape. Notably, not only do the flows not necessarily change across the border as manifested in the relatively stable ethnoscape, but they could also be contained, as observed in the case of the techno-mediascape.

Throughout this theoretically and empirically explorative study, we recognised the limitations of our research, including the small sample size and the unrepresentativeness of our participants of mainland students at large. Even though the limits of generalisability concerning this study’s findings must be taken seriously, this paper makes a significant contribution to the literature on ISM. Our study proposes a re-conceptualisation of the two-way horizontal or vertical mobility into a more fluid crisscrossing mobilities of people, ideas, techno-media and finance. Our approach complements the predominantly acculturation-centric perspectives, which whilst illuminating, ultimately under-describe the impact of the interaction of global flow dynamics on students. Whilst this present research endeavours to offer a more nuanced evaluation of student impacts due to these global flows, the intricate and inexorable process of acculturation is also, and perhaps unavoidably, evidenced in our own study.
